# A randomised controlled trial of a mindfulness intervention for men with advanced prostate cancer

**DOI:** 10.1186/1471-2407-13-89

**Published:** 2013-02-26

**Authors:** Suzanne K Chambers, David P Smith, Martin Berry, Stephen J Lepore, Elizabeth Foley, Samantha Clutton, Robert McDowall, Stefano Occhipinti, Mark Frydenberg, Robert A Gardiner

**Affiliations:** 1 Griffith Health Institute, Griffith University, 4222, Gold Coast, QLD, Australia; 2 Cancer Council Queensland, Brisbane, Australia; 3 Prostate Cancer Foundation of Australia, Sydney, Australia; 4 Australian and New Zealand Urogenital and Prostate Cancer Trials Group, Sydney, Australia; 5 Cancer Council NSW, Sydney, Australia; 6 University of New South Wales, Sydney, Australia; 7 Department of Public Health, Temple University, Philadelphia, USA; 8 Mind Potential, Sydney, Australia; 9 School of Psychology, Griffith University, Brisbane, Australia; 10 Department of Surgery, Monash University, Melbourne, Australia; 11 Royal Melbourne Hospital, Melbourne, Australia; 12 University of Queensland Centre for Clinical Research, University of Queensland, Brisbane, Australia; 13 Department of Urology, Royal Brisbane and Women’s Hospital, Brisbane, Australia

**Keywords:** Prostate cancer, Randomised controlled trial (RCT), Supportive care, Mental health, Psychological distress, Quality of life, Health outcomes

## Abstract

**Background:**

Prostate cancer is the most common male cancer in developed countries, and in Australia approximately one-fifth of men with prostate cancer have advanced disease. By comparison to men with localised prostate cancer, men with advanced disease report higher levels of psychological distress; poorer quality of life; and have an increased risk of suicide. To date no psychological intervention research specifically targeting men with advanced prostate cancer has been reported. In this paper we present the protocol of a current randomised controlled trial to assess the effectiveness of a professionally-led mindfulness-based cognitive therapy (MBCT) group intervention to improve psychological well-being in men with advanced prostate cancer.

**Methods/design:**

Ninety-five men per condition (190 men in total) will be recruited through clinicians in the Australian and New Zealand Urogenital and Prostate Cancer Trials Group and in major treatment centres in Queensland, New South Wales, Victoria and Western Australia. Patients are randomised to: (1) tele-based MBCT intervention or (2) patient education. A series of previously validated and reliable self-report measures will be administered to men at four time points: baseline/recruitment, and at 3, 6, and 9 months after recruitment and intervention commencement. Engagement with the principles of mindfulness and adherence to practice will be included as potential mediators of intervention effect. Primary outcomes are anxiety, depression and cancer-specific distress. Secondary outcomes are health-related quality of life (QoL) and benefit finding. Disease variables (e.g. cancer grade, stage) will be assessed through medical records.

**Discussion:**

This study will address a critical but as yet unanswered research question: to identify an effective way to reduce psychological distress; and improve the quality of life for men with advanced prostate cancer.

**Trial registration:**

http://ACTRN12612000306819

## Background

Prostate cancer is the most common male cancer in developed countries (excluding non-melanoma skin cancer). In 2007, 19,403 Australian men were diagnosed with prostate cancer [1]. Approximately 10-15% of men with prostate cancer have locally advanced or metastatic prostate cancer at diagnosis [2] and a further 20-40% of men with localised cancer at diagnosis experience recurrence or progression after treatment [3]. Men with advanced prostate cancer face additional physical and psychological challenges compared to men with localised disease. The iatrogenic effects of hormonal ablation, the main treatment for advanced disease, include mood disturbance, cognitive impairment, fatigue, and sexual dysfunction [4]. By comparison to men with localised prostate cancer, men with advanced disease report higher levels of psychological distress and poorer quality of life (QoL) [5,6]; and have an increased risk of suicide [7,8]. Hence research into psychological intervention to maximise psychological adjustment is crucial for these men.

To date, no psychological intervention research specifically targeting men with advanced prostate cancer has been reported [9]. In this study we propose using the cognitive behavioural approach of Mindfulness-Based Cognitive Therapy (MBCT) as relevant to this patient group. Mindfulness involves open awareness of current experience and the intention to observe habits of reacting to difficulties as they arise. Over time, this practice leads the person to gain the ability to be less reactive to difficult experiences and approach equanimity regarding the content of the illness experience, including negative emotions and thoughts. MBCT specifically targets the cognitive processes associated with depression by encouraging participants to disengage from reactive and ruminative states of mind, such as those that are commonly reported by cancer patients [10,11]. Qualitative studies of cancer patients who have taken part in mindfulness courses have identified positive changes in acceptance, self-control, personal growth, shared experience and self-regulation as outcomes of mindfulness practice [12,13]. In a pilot study of group MBCT conducted with 19 men with advanced prostate cancer, men reported significant changes in both general psychological and cancer-specific distress [14]. Qualitative data revealed that acceptance of and learning from other group members were key aspects of the group context that contributed to acceptance of progressive disease. Thus, in the context of MBCT the group setting appears important as a contributor to acceptance of cancer through peer learning and modelling.

Accordingly, in this trial we apply a tele-based MBCT group intervention to decrease anxiety and depression and cancer-specific distress in men with advanced prostate cancer.

## Methods/design

The study has two arms: 1) MBCT delivered by teleconference over eight weekly sessions and (2) patient education.

It is hypothesised that 3, 6 and 9 months after recruitment and commencement of the intervention:

1. Relative to men who receive patient education, men who receive MBCT will have lower anxiety and depression.

2. Relative to men who receive patient education, men who receive MBCT will have lower cancer-specific distress.

3. Relative to men who receive patient education, men who receive MBCT will have higher mindfulness.

4. Intervention-driven improvements in psychological outcomes will be mediated by mindfulness skills.

### Group condition

#### *Patient Education*

Patient education consists of the man’s standard medical management and a package containing existing evidence-based patient education materials.

#### *Mindfulness-Based Cognitive Therapy*

The Mindfulness-Based Cognitive Therapy group intervention (MBCT) follows a cancer-specific manual based on Segal, Williams and Teasdale’s [15] manual for MBCT, with novel specific components developed for men with advanced prostate cancer in our pilot study [14]. The sessions are facilitated by health professionals with experience in oncology and professional training in MBCT. The program has been further developed to be suitable for telephone delivery. For example, facilitators have been trained to use explicit communication such as encouraging group members to say their name before contributing, listen closely to the tone of participants' voices, give extra time for responses, and have explicit rules for how the group will interact. Each session runs for 1.25-h and only short (up to 15 min) meditations are provided during the group phone sessions to support group engagement and alleviate practical concerns such as holding the telephone receiver. As well, the material in participant workbooks has been elaborated to provide session plans, so participants can navigate phone sessions more easily, and interactive worksheets are provided to help keep group discussions on task. The program includes eight weekly group teleconferences, and each participant has an individual introductory call with their facilitator to allow them to connect with the facilitator, prepare them for the program and to enhance motivation. Participants are provided with a handbook summarising each weekly session; self-help materials including Jon Kabat Zinn’s Full Catastrophe Living [16]; and an audio recorded meditation on compact disc. Daily home practice of mindfulness meditation is strongly encouraged; with participants asked to engage in one of the four 35 min practices depending on the stage of the course (the body scan, moving meditation, mixed mindfulness meditation, silent practice with bells at 5 min intervals). Finally, as in our pilot study, peer interaction is directed towards support for the learning of mindfulness skills and mutual support in facing the challenges of advanced prostate cancer.

### Participants

**Figure 1:**
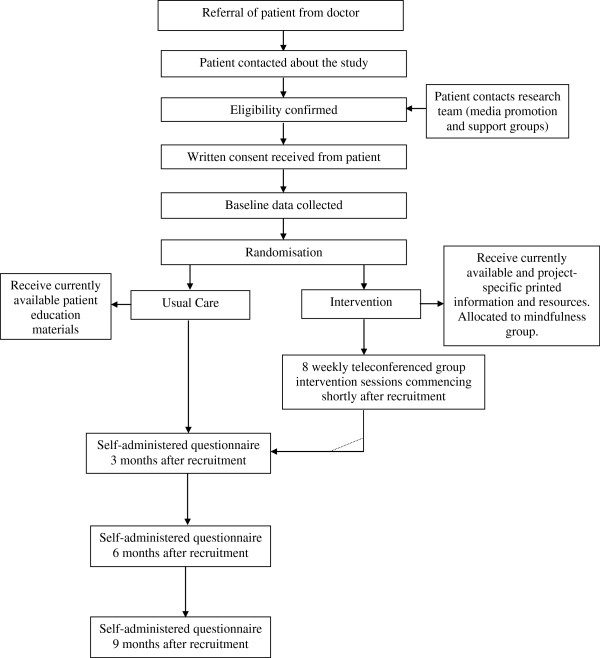
Flowchart of recruitment, intervention and assessment.

Recruitment is being undertaken through clinicians in the Australian and New Zealand Urogenital and Prostate Cancer Trials Group and in major treatment centres in Queensland, New South Wales, Victoria and Western Australia. Other recruitment avenues include the distribution of information through prostate cancer support groups and media promotion. The research team who contact potential participants after referral to the study obtains informed written consent. Figure 1 illustrates the recruitment, intervention and data collection process.

Inclusion criteria are that the men must: (1) have proven metastatic disease *or* castration resistant biochemical progression; (2) be able to read and speak English; (3) have no previous history of head injury, dementia or psychiatric illness; (4) have no other concurrent cancer; (5) have phone access.

Approximately 190 men will be recruited to the study (allowing 30% attrition from treatment; 65 men in each condition will complete final assessments). This sample size would comfortably exceed 80% power to detect a moderate to large effect over four assessment points.

### Study integrity

Ethical approval has been obtained from the Griffith University Human Research Ethics Committee (Approval: PSY/15/12/HREC) and Metro-North – The Prince Charles Hospital Human Research Ethics Committee (HREC/12/QPCH/101). The study design is guided by the CONSORT criteria [17]. Randomisation to study condition occurs following the completion of baseline assessment. Assessments are by self-report pen and paper measures and project staff tracking assessments are blinded to condition where possible. Randomisation occurs in blocks of 14, with each condition randomly generated 7 times within each block to ensure an unpredictable allocation sequence with equal numbers of men in each condition at the completion of each block; and sufficient men to form a tele-based group (of 7) in the MBCT condition. Randomisation occurs within Queensland-Western Australia and New South Wales-Victoria dyads to coincide with a two-stage commencement of recruitment. The randomisation sequence is undertaken by the project manager and concealed from investigators. The group sessions are audiotaped with 15% reviewed to ensure adherence to an MBCT approach. Analyses will be conducted on the basis of intention to treat.

### Measures

A series of previously validated and reliable self-report measures are administered by mail to men at four time points: baseline/recruitment and at 3, 6, and 9 months after recruitment and intervention commencement. Mindfulness skills are included as potential mediators of intervention effect. Primary outcomes are anxiety, depression and cancer-specific distress. Secondary outcomes are global QoL and benefit finding. Disease variables (e.g. cancer grade, stage, time since diagnosis, time since treatment) will be assessed through medical records review.

#### Mediators

Mindfulness: The Five Facet Mindfulness Questionnaire (FFMQ) [18] is being used to measure the participants’ engagement with the principles of mindfulness.

Adherence to Practice: Adherence to mindfulness practice is assessed by participants completing a daily home practice form.

#### Primary outcome variables

Anxiety and Depression: The Brief Symptom Inventory-18 [19] is providing a global measure of current psychological distress with subscale scores for anxiety, depression, and somatisation.

Cancer-Specific Distress: The Impact of Events Scale (IES) [20,21] and the PSA Anxiety subscale of the Memorial Anxiety Scale for Prostate Cancer (MAX-PC) [22] is being used to measure men’s cancer-specific distress.

#### Secondary outcome variables

Quality of Life: The Functional Assessment of Cancer Therapy – Prostate (FACT-P) [23] is being used to assess the men’s perceived global quality of life across 5 domains: physical, social/family, emotional, functional well-being, and prostate cancer-specific concerns.

Benefit Finding: Positive adjustment is being measured with the Posttraumatic Growth Inventory assessing perceived positive outcomes resulting from a diagnosis of cancer [24].

### Statistical analyses

The study is a two-condition randomised controlled trial with repeated measures across time and continuous outcome variables. The analysis of longitudinal differences in outcome will be by multilevel (mixed) modelling (MLM). These procedures allow the testing of typical group level predictions such as Hypotheses 1 to 3 that men in the intervention condition will have better outcomes than the patient education group. However, by incorporating the hierarchical structure of assessment points nested within individual men they further permit the true assessment of individual change in psychological outcomes and of potential mediators of such change (Hypothesis 4). Consequently (and unlike traditional approaches), this model deals with the heterogeneity of responses, such as that expected in the outcomes of the proposed study, by allowing such variation as random effects within the model. MLM has the advantage of allowing use of all available data points, which maximizes power to detect effects and reduces bias owing to missing data in longitudinal studies.

## Discussion

The study will provide recommendations about effective psychological interventions to reduce anxiety, depression and cancer-specific distress in men with advanced prostate cancer. To our knowledge, to date no psychological intervention studies have targeted men with advanced prostate cancer specifically; or trialled remotely delivered mindfulness interventions in cancer populations [9]. This research will overcome these limitations. If proven effective, the intervention will be able to be utilised in a range of settings including broad reach tele-health support programs; and through support services and support groups internationally. This means that project outputs will be immediately translatable into practice to reduce psychological distress and improve the quality of life of men with advanced prostate cancer.

## Competing interests

The authors declare that they have no competing interests.

## Authors’ contributions

SKC, DS and MB developed the study concept and aims and initiated the project. SL, EF, SC, RM, SO, MF and RAG assisted in further development of the protocol. SKC was responsible for drafting the manuscript. SKC, DS, EF, SC, RM and SO will implement the protocol and oversee collection of the data. All authors contributed to the final manuscript.

## Pre-publication history

The pre-publication history for this paper can be accessed here:

http://www.biomedcentral.com/1471-2407/13/89/prepub
